# Inhibition of Bruton's TK regulates macrophage NF‐κB and NLRP3 inflammasome activation in metabolic inflammation

**DOI:** 10.1111/bph.15182

**Published:** 2020-08-26

**Authors:** Gareth S.D. Purvis, Massimo Collino, Haidee Aranda‐Tavio, Fausto Chiazza, Caroline E. O'Riordan, Lynda Zeboudj, Shireen Mohammad, Debora Collotta, Roberta Verta, Nicolas E.S. Guisot, Peter Bunyard, Magdi M. Yaqoob, David R. Greaves, Christoph Thiemermann

**Affiliations:** ^1^ William Harvey Research Institute Queen Mary University of London London UK; ^2^ Sir William Dunn School of Pathology University of Oxford Oxford UK; ^3^ Department of Drug Science and Technology University of Turin Turin Italy; ^4^ Redx Pharma Macclesfield UK; ^5^ Centre for Diabetic Kidney Disease Bart's and The London Hospital London UK

**Keywords:** Bruton's tyrosine kinase, diabetes, drug repurposing, macrophage, metabolic inflammation, NF‐kB, NLRP3

## Abstract

**Background and Purpose:**

There are no medications currently available to treat metabolic inflammation. Bruton's tyrosine kinase (BTK) is highly expressed in monocytes and macrophages and regulates NF‐κB and NLRP3 inflammasome activity; both propagate metabolic inflammation in diet‐induced obesity.

**Experimental Approach:**

Using an *in vivo* model of chronic inflammation, high‐fat diet (HFD) feeding, in male C57BL/6J mice and *in vitro* assays in primary murine and human macrophages, we investigated if ibrutinib, an FDA approved BTK inhibitor, may represent a novel anti‐inflammatory medication to treat metabolic inflammation.

**Key Results:**

HFD‐feeding was associated with increased BTK expression and activation, which was significantly correlated with monocyte/macrophage accumulation in the liver, adipose tissue, and kidney. Ibrutinib treatment to HFD‐fed mice inhibited the activation of BTK and reduced monocyte/macrophage recruitment to the liver, adipose tissue, and kidney. Ibrutinib treatment to HFD‐fed mice decreased the activation of NF‐κB and the NLRP3 inflammasome. As a result, ibrutinib treated mice fed HFD had improved glycaemic control through restored signalling by the IRS‐1/Akt/GSK‐3β pathway, protecting mice against the development of hepatosteatosis and proteinuria. We show that BTK regulates NF‐κB and the NLRP3 inflammasome specifically in primary murine and human macrophages, the *in vivo* cellular target of ibrutinib.

**Conclusion and Implications:**

We provide “proof of concept” evidence that BTK is a novel therapeutic target for the treatment of diet‐induced metabolic inflammation and ibrutinib may be a candidate for drug repurposing as an anti‐inflammatory agent for the treatment of metabolic inflammation in T2D and microvascular disease.

## Graphical abstract

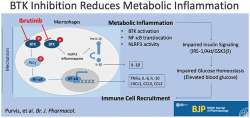

**Abbreviations**
ACR: albumin to creatinine ration
BMDM: bone marrow derived macrophage
BTK: Bruton's tyrosine kinase
CLL: chronic lymphatic leukaemia
EMA: European Medicines Agency
FDA: U.S. Food and Drug Administration
HFD: high fat diet
hMoDM: human monocyte derived macrophage
NLPR3: NLR family pyrin domain containing 3
OGTT: oral glucose tolerance test
T2D: Type 2 diabetes
TLR: toll‐like receptor

**What is already known**

- BTK is a druggable target, with FDA approved medications available.

**What does this study add**

- BTK expression and activation are increased in male C57BL/6J mice fed a high‐fat diet.
- Inhibition of BTK in HFD‐fed mice decreased macrophage NF‐κB and NLRP3 inflammasome activity.

**What is the clinical significance**

- We have identified BTK as a new pathway that is activated in metabolic inflammation.
- Ibrutinib may represent a novel candidate for drug repurposing for the treatment of metabolic inflammation.

## 1 INTRODUCTION

The worldwide prevalence of obesity has doubled since 1980 with over 1.9 billion people being considered overweight or obese (Swinburn et al., 2011). Diet‐induced obesity induces a state of chronic metabolic inflammation (Saltiel & Olefsky, 2017). The control of energy and metabolism in tissues is directed largely by innate immune cells, such as macrophages leading to the production of soluble effector molecules including cytokines and chemokines (Tanti, Ceppo, Jager, & Berthou, 2013). This state of “low‐grade” chronic inflammation predisposes individuals to metabolic syndrome and the associated co‐morbidities that develop over time including insulin resistance, Type 2 diabetes (T2D), cardiovascular disease and non‐alcoholic fatty liver disease (Lusis, Attie, & Reue, 2008) (Esposito & Giugliano, 2004).

Immune cell accumulation specifically from the myeloid linage coupled with the activation of the pathways including those involving NF‐κB and the NLRP3 inflammasome have been heavily implicated in orchestrating the inflammatory response in T2D (Arkan et al., 2005) (Vandanmagsar et al., 2011). Pharmacological inhibition or genetic deletion of components of the NF‐κB pathway (i) prevents the development of HFD‐induced insulin resistance (Chiazza et al., 2015) (Benzler et al., 2015) and (ii) slows the progression of microvascular disease. Equally, inhibition or genetic deletion of key components the NLRP3 inflammasome reduced systemic inflammation in models of diet‐induced obesity and protected again the development of peripheral insulin resistance in mice (Chiazza et al., 2016).

Currently, there are no medicines for T2D that target metabolic inflammation and/or prevent the development of microvascular complications. One option for generating new treatments for rapid patient benefit is to repurpose existing U.S. Food and Drug Administration (FDA) or European Medicines Agency (EMA) approved drugs. Exploring existing medications that have under‐appreciated anti‐inflammatory effects for new indications could be a time‐ and cost‐effective approach to prevent and/or treat the development of microvascular complications of T2D. Anti‐inflammatory agents have been shown to be powerful tools to study disease pathophysiology in preclinical models of T2D. However, to date, little translational research as followed up on these successes.

Germline mutations in Bruton's tyrosine kinase (BTK) have been implicated in the primary immunodeficiency disease X‐linked agammaglobulinaemia, through its essential role in B lymphocyte development (Nyhoff et al., 2018). The first BTK inhibitor to be FDA approved for clinical use was ibrutinib for the treatment of chronic lymphatic leukaemia (CLL) (Davids & Brown, 2012). However, BTK has an additional role in regulating inflammation, being highly expressed in monocytes/macrophages (Ito et al., 2015). BTK has a proposed role in signal transduction downstream of several toll‐like receptors (TLR) which results in reduced activation of NF‐κB (Ní Gabhann et al., 2014) and also regulates NLRP3 inflammasome assembly in macrophages (Ito et al., 2015). Importantly, BTK is not expressed in hepatocytes or adipocytes, the other cell types responsible for peripheral insulin resistance (Uhlén et al., 2015) and so has a specific role in macrophages, the key cell type that drives the production of pro‐inflammatory mediators that leads to the development of metabolic syndrome and insulin resistance.

Ibrutinib has therapeutic utility in a number of preclinical models of human disease; however, most of these models have a strong B‐cell component (Honigberg et al., 2010; Chalmers et al., 2018). In the present study, we investigated whether treatment with ibrutinib reduces metabolic inflammation in a murine model of HFD‐induced obesity. HFD feeding results in an increase in macrophage number in peripheral tissues including the liver, adipose tissue, and kidney. We hypothesized that ibrutinib treatment would target the cells, which express high levels of BTK, namely, monocytes and macrophages, reducing their pro‐inflammatory activity *in vivo.* We predicted that inhibition of BTK signalling would result in decreased activation of both NF‐κB and the NLRP3 inflammasome in mice fed a HFD. We report for the first time that treatment with a BTK inhibitor results in reduced metabolic inflammation and improved function in the liver, adipose tissue, and the kidney.

## 2 METHODS

### 2.1 Animals and human studies

All animal care and experimental protocols in this study was in accordance with the Home Office guidance on Operation of Animals (Scientific Procedures Act 1986) and were approved by the Animal Welfare Ethics Review Board (AWERB) of Queen Mary University of London and the University of Oxford. The study was performed under licences issued by the Home Office PPL:70/8052 and P144E44F2. Animal studies are reported in compliance with the ARRIVE guidelines (Kilkenny et al., 2010) and with the recommendations made by the British Journal of Pharmacology.

#### 2.1.1 High‐fat diet induced insulin resistance

Ten weeks old male C57BL/6J mice purchased from Charles River Ltd, housed in the same unit under conventional housing conditions at 25 ± 2°C, were randomly assigned either normal diet (chow) (7% simple sugars, 3% fat, 50% polysaccharide, 15% protein) or high‐fat diet (HFD) (D12331 diet, Research Diet Inc., USA). All mice had access to food and water ad libitum. After 6 weeks of dietary manipulation, mice were randomly assigned to a treatment group receiving either ibrutinib (3 or 30 mg·kg^−1^, in vehicle) or vehicle alone (5% DMSO, 30% cyclodextrin) for 5 days per week for 6 weeks by oral gavage. The doses used here are within the range that have previously been reported to produce selective inhibition of BTK, in a short‐term model of cerebral ischaemia (Ito et al., 2015) and in a chronic model of arthritis (Honigberg et al., 2010). One week prior to the end of the experiment, an oral glucose tolerance test was performed. 24 h before the end of the experiment, mice were placed in metabolic cages and urine collected. Blood was collected by cardiac puncture under general anaesthesia (Ketamine/Xylazine [100 mg·kg^−1^ and 10 mg·kg^−1^; i.p.] [Centaur Services, UK]).

#### 2.1.2 XID mice

XID mice (B6.CBA‐*Btk* ^*xid*^/AllmJ) (Lindsley, Thomas, Srivastava, & Allman, 2007) are an inbred strain on the CBA background purchased from The Jackson Laboratory (#009361). They have a point mutation rendering the kinase domain of BTK inactive. Specifically, there is a C to T substitution at coding nucleotide 82, which alters the amino acid sequence; substituting an arginine for cysteine. The substitution is in a conserved PH domain and blocks the activation of the kinase (Rawlings et al., 1993) preventing BTK phosphorylation at Tyr^223^, which is a key activating site. Importantly, ibrutinib binds irreversibly to Cys^481^, also in the active site of the kinase domain and inhibits auto‐phosphorylation of Tyr^223^, thus blocking BTK activity.

### 2.2 Cell culture

#### 2.2.1 Macrophage reporter cell line (RAW Blue)

A commercially available reporter macrophage cell line (RAW Blue cells; InvivoGen, San Diego, CA; RRID:CVCL_X594) was used: Briefly, cells were derived from murine RAW 264.7 macrophages with chromosomal integration of a secreted embryonic alkaline phosphatase (SEAP) reporter construct, induced by NF‐κB and activator protein 1 (AP‐1) transcriptional activation. Cells were grown to 80% confluence in T‐25 culture flasks in DMEM containing 4.5 g·L^−1^ glucose, heat‐inactivated 10% FBS, 2 mM l‐glutamine, and 200 μg·ml^−1^ Zeocin antibiotic (InvivoGen) at 37°C in 5% CO_2_. To minimize experimental variability, only cells with fewer than five passages were used. Cells were plated at 0.95 × 10^5^ per well. After experimental treatment, 20 μl of cell supernatants was added to 180 μl of QuantiBlue substrate (InvivoGen) and incubated at 30°C for 60 min, plate was then read at an OD at 655 nm (OD_655_) on a microplate spectrophotometer (PherastarFSX, BMG Lab, UK).

#### 2.2.2 Murine bone marrow‐derived macrophages (BMDMs)

Bone marrow‐derived macrophages were generated as previously described (Recio et al., 2018). Briefly, fresh bone marrow cells from tibiae and femurs of male C57BL/6 or XID mice aged 8–10 weeks were cultured in DMEM containing 4.5 g·L^−1^ glucose, 2 mM l‐glutamine, 50 units·ml^−1^ penicillin and 50 μg·ml^−1^ streptomycin, 10% heat‐inactivated FBS, 10% L929 cell‐conditioned media (as a source of macrophage colony‐stimulating factor) and for 7 days. Bone marrow cells were seeded into 8 ml of medium in 90 mm non‐tissue culture treated Petri dishes (ThermoFisher Scientific, Sterilin, UK). On Day 5, an additional 5 ml of medium was added. Gentle scraping was used to lift cells. BMDMs were then counted and suspended in FBS free media at the desired cell concentration.

#### 2.2.3 Human monocyte‐derived macrophages (hMoDMs)

Peripheral blood mononuclear cells (PBMC) were purified from leukocyte cones from healthy volunteers with informed consent (NHSBTS, Oxford, UK) by density centrifugation over Ficoll‐Paque PLUS (Sigma). The PBMC layer was carefully harvested and then washed twice with PBS. Monocytes were then isolated from the PBMCs by negative selection using magnetic beads (Miltenyi Biotec, Bergisch Gladbach, Germany). Cells were maintained in RPMI 1640 medium supplemented with 1% human serum, 50 ng·ml^−1^ macrophage colony stimulating factor (hM‐CSF, BioLegend), 50 units·ml^−1^ penicillin and 50 μg·ml^−1^ streptomycin for 7 days.

### 2.3 Caspase 1 assay

Caspase 1 activity was measured using commercially available kits (Caspase 1 Glo inflammasome assay, Promega). Briefly, BMDM (2.5 × 10^4^) were seeded on half area, white walled, 96‐well multiwell plates. BMDM were pretreated with ibrutinib for 1 h prior to 8 h LPS (100 ng·ml^−1^) stimulation. To assay caspase 1 activity, ATP (5 mM) was added for the last 60 min. Samples (20 μl ) of cell supernatant were then then incubated with 40 μM Z‐WEHD‐aminoluciferin (120 μM) MG‐132 inhibitor in Caspase‐Glo® 1 Buffer for 1 h. The luminescence was read with a microplate spectrophotometer (PherastarFSX, BMG Lab).

### 2.4 mRNA expression

Mouse tissues and cultured cells were harvested into TRIzol reagent (Life Technologies) and total RNA extracted. RNA concentration and quality was determined with a ND‐1000 spectrophotometer (Nano Drop Technologies, Wilmington, USA). cDNA was synthesized from 1,000 ng RNA using the QuantiTect Reverse Transcription kit (Qiagen, Manchester, UK) according to the manufacturer's instructions. Real‐time quantitative PCR was performed using Sybr Select gene expression master mix (Life Technologies) in the StepOnePlus™ thermal cycler (Applied Biosystems). Primers were purchased from Qiagen (*il1β*, *il18*, *tnfα*, *il6*, *il10*, *cxcl1*, *ccl2*, *ccl5*, *btk*, *CD68*, *cd38*, *screb*, *fasn*, *ucp2*, *ctp*, *lcad*, *scd1*, and *βactin*). Cycle threshold values were determined by the StepOne software, and target gene expression was normalized to housekeeping gene (*β‐actin* or *18S*).

### 2.5 Quantification of cytokine level

BMDM or hMoDM (1.5 × 10^6^) were seeded in 6‐well plates. BMDM or hMoDM were pretreated with ibrutinib for 1 h prior to stimulation with LPS (100 ng·ml^−1^) for 8 h. Culture medium was adjusted to 5 mM ATP in the last 60 min to allow secretion of IL‐1β.

#### 2.5.1 ELISA

Measurement of the protein levels of IL‐1β, TNFα and IL‐6, CXCL1, CCL2, and CCL5, secreted into cell supernatants from 1.5 × 10^6^ murine BMDMs was performed by ELISA (R&D Systems) according to the manufacturer's instructions.

#### 2.5.2 Bead assay

Measurement of protein levels of secreted IL‐1β, TNFα, and IL‐6 in cell supernatants from 1.5 × 10^6^ human MoDM's was performed by Legend Plex multi‐analyte assay kit (BioLegend) according to the manufacturer's instruction. Data were acquired using a BD Fortessa X20 cytometer (BD Biosciences) and analysed using LegendPlex Software (BioLegend).

### 2.6 Flow cytometry

Cells were washed in FACS buffer (0.05% BSA, 2 mM EDTA in PBS pH 7.4) blocked using anti CD16/32 (Biolegend; RRID:AB_312801) (for 10 min at 4°C, followed by antibody staining for the surface markers BV421‐conjugated CD11b (BioLegend; RRID:AB_10897942), PE‐conjugated CD14 (BioLegend; RRID:AB_314187) with appropriate isotype controls. Data were acquired using a BD Fortessa X20 cytometer (BD Biosciences; RRID:SCR_013311) and then analysed using FlowJo (Tree Star Inc, USA; RRID:SCR_008520) software.

### 2.7 Western blot

The immuno‐related procedures used in this study comply with the recommendations made by the British Journal of Pharmacology (Alexander et al., 2018). Tissues (liver and kidney) were lysed, as previously described (Purvis et al., 2017). Briefly, samples (100 mg) of of snap frozen tissue was suspended in 0.3 ml of RIPA buffer containing phosphatase and protease inhibitors (Sigma, UK) for 30 mins and cells lysed by mechanical disruption, then centrifuged at 10,000g for 15 mins at 4 C and the supernatant collected, Protein concentration was determined by using a BCA protein assay kit (ThermoFisher Scientific). Total cell protein (30 mg) was added to 4× Laemmli buffer (250 mM Tris–HCl, pH 6.8, 8% SDS, 40% glycerol, 0.004% bromophenol blue, 20% β‐mercaptoethanol) and heated at 95°C for 5 min. Samples were then resolved on SDS‐PAGE gels and transferred onto Hybond ECL nitrocellulose membranes (GE Healthcare, Buckinghamshire, UK). Membranes were blocked with 5% BSA (Sigma‐Aldrich) in TBS‐Tween for 1 h at RT and then incubated with the primary antibody, mouse anti‐Ser^473^‐Akt (Cell Signaling Technology Cat#4051; RRID:AB_331159), rabbit anti‐total Akt (Cell Signaling Technology Cat#9272; RRID:RRID:AB_329827), rabbit anti‐Ser^307^‐IRS1 (Cell Signaling Technology Cat# 2385, RRID:AB_330363), mouse anti‐total IRS1 (Cell Signaling Technology Cat# 3194, RRID:AB_561124), rabbit anti‐total BTK (Abcam Cat# ab51204, RRID:AB_868519), rabbit anti‐Tyr^223^ BTK (Abcam Cat# ab51210, RRID:AB_873714), rabbit anti‐total PLCγ (Cell Signaling Technology Cat# 3860, RRID:AB_1196659), rabbit anti‐Tyr^1217^ PLCγ (Cell Signaling Technology Cat# 3871, RRID:AB_2299548), rabbit anti‐phospho‐Ser^9^‐GSK‐3β (Cell Signaling Technology Cat# 9322, RRID:AB_2115196), rabbit anti‐total GSK‐3β (Cell Signaling Technology Cat# 9315, RRID:AB_490890), mouse anti‐Ser^32/36^ IKBα (Cell Signaling Technology Cat# 4814, RRID:AB_390781), mouse anti‐total IKBα (Cell Signaling Technology Cat# 9242, RRID:AB_331623), rabbit anti‐Ser^176/180^‐IKKα/β (Cell Signaling Technology Cat# 2697, RRID:AB_2079382), rabbit anti‐total IKKα/β (Cell Signaling Technology Cat# 2370, RRID:AB_2122154), rabbit anti‐NF‐κB (Cell Signaling Technology Cat# 8242, RRID:AB_10859369), ASC (Cell Signaling Technology Cat# 67824, RRID:AB_2799736), NLRP3 (Cell Signaling Technology Cat# 15101, RRID:AB_2722591), gasdermin D (Cell Signaling Technology Cat# 93709, RRID:AB_2800210), IL‐1β (Cell Signaling Technology Cat# 12242, RRID:AB_2715503) and mouse anti‐caspase 1 p20 (AdipoGen Cat# AG‐20B‐0042, RRID:AB_2490248) diluted 1:1,000 in 2% BSA/TBS‐Tween overnight at 4°C. Next, membranes were incubated with an HRP‐conjugated anti‐rabbit (Cell Signaling Technology Cat# 7074, RRID:AB_2099233) or anti‐mouse (Cell Signaling Technology Cat# 7076, RRID:AB_330924) secondary antibody diluted 1:10,000 in 2% BSA for 1 h at room temperature. Protein bands were visualized by incubating the membranes for 5 min with Amersham ECL prime and subsequent exposure to X‐ray film over a range of exposure times. Rabbit anti‐β‐actin (Cell Signaling Technology Cat# 4970, RRID:AB_2223172) and rabbit anti‐histone 3 (Cell Signaling Technology Cat# 4499, RRID:AB_10544537) were used as loading control. For successive antibody incubations using the same membrane bound antibodies were removed with stripping buffer (ThermoFisher Scientific).

### 2.8 Histology

#### 2.8.1 Immunofluorescence

For p65 expression and location detection by immunofluorescence, cells placed in a 4‐well chamber‐slide were fixed (4% paraformaldehyde) for 10 min at room temperature, permeabilized (0.5% Triton X‐100 in PBS) for 10 min at 4°C, blocked with 4% BSA/PBS containing 6% donkey serum, and incubated with mouse anti‐p‐65 antibody diluted (Cell Signaling Technology Cat# 8242, RRID:AB_10859369) 1:100 in 4% BSA/PBS, overnight at 4°C. Cells were then incubated with donkey anti‐mouse Alexa Fluor 488 (Thermo Fisher Scientific Cat# A‐11034, RRID:AB_2576217) (diluted 1:500 in 4% BSA/PBS for 1 hour at room temperature. DAPI was used for nuclear counterstaining. The slide was mounted with Fluormount‐G®, and cells were visualized using a confocal microscope.

#### 2.8.2 Oil Red O staining

Frozen liver samples were embedded in OCT and cut in 10 μm sections. Sections were brought to room temperature, fixed with 10% buffer formalin for 5 min, washed with 60% isopropanol, then saturated with Oil Red O (1% w/v, 60% isopropanol) for 15 min, washed in 60% isopropanol and rinsed in distilled water. Then sections were then mounted in aqueous mounting medium with coverslips. Images were acquired using a NanoZoomer Digital Pathology Scanner (Hamamatsu Photonics K.K., Japan) and analysed using the NDP Viewer software.

#### 2.8.3 Periodic acid Schiff's staining

Kidney samples were obtained at the end of the experiment and fixed in 10% neutral‐buffered formalin for 48 h, and histology staining was performed. Briefly, kidney tissue was embedded in paraffin and processed to obtain 4 μm sections. After deparaffinization, sections were rehydrated through graded alcohol to distilled water. The sections were then incubated to saturation in periodic acid Schiff's solution (Sigma, UK) for 30 min and washed in distilled water. Then sections were then dehydrated through graded alcohols and cleared before mounting with coverslips. Images were acquired using a NanoZoomer Digital Pathology Scanner and analysed using the NDP Viewer software (Purvis et al., 2019).

#### 2.8.4 Immunohistochemistry

Kidney sections cut at 4 μm were deparaffinized to PBS. Antigen retrieval was performed by in citrate buffer (pH 6.0) for 15 min. Once cooled, sections were incubated with 3% H_2_O_2_ for 20 min to inactivate endogenous peroxidases (Dako EnVision+ System‐HRP‐DAB, K4010) and subsequently treated with 10% normal goat serum (Dako, UK) to reduce non‐specific absorption. Sections were subsequently incubated at 37°C for 1 h with the following primary antibodies: anti‐F4/80 (Abcam Cat# ab111101, RRID:AB_10859466) washed with PBS and then incubated at room temperature for 30 min with labelled polymer‐HRP antibody (Dako EnVision+ System, HRP‐DAB). Sections were developed in DAB chromogen solution, and the reaction stopped by immersion of sections in water. Counterstaining was performed with Harris haematoxylin before sections were dehydrated and mounted in DPX mounting medium.

### 2.9 Data and statistical analysis

The data and statistical analysis comply with the recommendations of the *British Journal of Pharmacology* on experimental design and analysis in pharmacology (Curtis et al., 2018). All experiments were designed, where possible, to generate groups of equal size. Power calculations were used to estimate the group size based on an expected effect size of 30%. Where possible, blinding and randomisation protocols were used. All data in the text and figures are presented as mean ± standard error mean (SEM) of *n* observations, where *n* represents the number of animals studied (in vivo) or independent values, not technical replicates (in vitro). For western blot data, all data are represented as fold change to mean control (normal chow diet). All statistical analysis was calculated using GraphPad Prism 7 for Mac (GraphPad Software, San Diego, California, USA; RRID:SCR_002798). Statistical analysis was only undertaken for studies where each group size was at least *n* = 5. For western blot analysis, some representative data are shown where group size is less then *n* = 5. When the mean of two experimental groups were compared, a two‐tailed Students *t*‐test was performed. Normally distributed data without repeated measurements were assessed by a one‐way ANOVA followed by Bonferroni correction if the *F* value reached significance. In all cases a *P* < 0.05 was deemed significant.

### 2.10 Data and resource availability

The datasets generated during and/or analysed during the current study are available from the corresponding author upon reasonable request. No novel resources were generated during the current study.

### 2.11 Materials

Ibrutinib (Cambridge BioScience, UK); LPS (Lipopolysaccharides from Escherichia coli O111:B4, Sigma, UK), general anaesthetic used.

### 2.12 Nomenclature of targets and ligands

Key protein targets and ligands in this article are hyperlinked to corresponding entries in the IUPHAR/BPS Guide to PHARMACOLOGY (http://www.guidetopharmacology.org) and are permanently archived in the Concise Guide to PHARMACOLOGY 2019/20 (Alexander, Fabbro, et al., 2019a, b; Alexander, Kelly, et al., 2019a, b).

## 3 RESULTS

### 3.1 Treatment with ibrutinib reduces the diabetic phenotype in a murine model of HFD feeding

**FIGURE 1:**
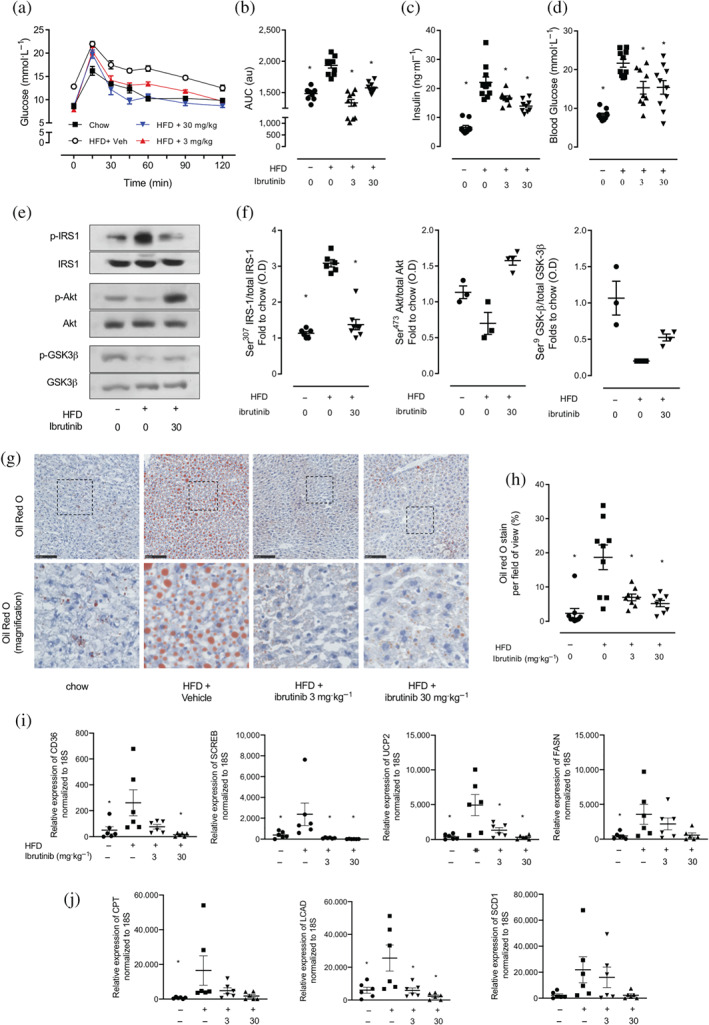
Ibrutinib treatment restored insulin signalling through IRS‐1/Akt/GSK‐3β in mice fed a HFD. (a) Oral glucose tolerance (OGTT) was assessed over 120 min, 1 week prior to harvest. (b) The AUC of OGTT was calculated for respective groups and used for statistical analysis. (c) Plasma insulin levels were measured in plasma isolated from whole blood at harvest. (d) Basal, non‐fasted, blood glucose was measured at week 11 1 h prior to harvest. Data shown are (in a) means ± SEM in b, c, d, with individual values; *n* = 9‐10 per group. ^*^ *P* < 0.05, significantly different from HFD + Veh; one‐way ANOVA with Bonferroni post hoc test. (e) Representative western blots for phosphorylation of Ser^307^ on IRS‐1 in the liver and normalized to total IRS‐1; for phosphorylation of Ser^473^ on Akt in the liver and normalized to total Akt; for phosphorylation of Ser^9^ on GSK‐3β in the liver and normalized to total GSK‐3β and (f) quantified using densitometry. Data shown are individual values with means ± SEM; *n* = 3–6 per group. ^*^ *P* < 0.05, significantly different from HFD + Veh; one‐way ANOVA with Bonferroni post hoc test. (g) Representative images of hepatic lipid deposition assessed by Oil Red‐O staining and (h) quantified. Scale bars measure 50 μm. Inset images are 4× digital zoom. (i) Relative gene expression of *CD36*, *SCREB*, *UCP2*, and *FASN* were assessed by qPCR and normalized to *18S*. (j) Relative gene expression of *CTP*, *LCAD*, and *SCD1* were assessed by qPCR and normalized to *18S.* and gene expression data. Data shown are individual values with means ± SEM; *n* = 6 per group. ^*^ *P* < 0.05, significantly different from HFD + Veh; one‐way ANOVA with Bonferroni post hoc test

C57BL/6J mice were fed either chow or HFD for 6 weeks and then treated with ibrutinib (3 or 30 mg·kg^−1^; p.o.; five times per week) or vehicle for a further 6 weeks. At the time of treatment, mice fed a HFD had developed a small, but significant, augmentation in oral glucose tolerance test (OGTT) (Figure S1A, B). Importantly, by week 12 of HFD feeding, control mice fed a HFD gained more weight than mice fed a chow diet, specifically they gained more fat mass (Table 1). Chronic treatment with ibrutinib (3 or 30 mg·kg^−1^) to chow or HFD‐fed mice did not alter calorific intake, weight gain or cause hepatocellular damage (Figure S1C–F; Table 1). When compared to mice fed a chow diet, mice fed a HFD and treated with vehicle exhibited a significant elevation in fasting blood glucose (values at time=0; Figure 1a)). Mice fed a HFD and treated with either 3 or 30 mg·kg^−1^ ibrutinib had significantly lower fasting blood glucose, compared with that in mice fed a HFD and treated with vehicle (values at time=0; Figure 1a). When compared to mice fed a chow diet that underwent an oral glucose tolerance test (OGTT), mice fed a HFD exhibited a significant and prolonged elevation in blood glucose levels (Figures 1a, b). HFD mice also had elevated terminal plasma insulin levels (Figure 1c) and non‐fasting blood glucose (Figure 1d). Mice fed a HFD and treated with either 3 or 30 mg·kg^−1^ ibrutinib displayed no significant change in OGTT when compared to chow fed mice (Figure 1a,b), and significantly lower plasma insulin levels (Figure 1c) and non‐fasting blood glucose (Figure 1d) when compared to mice fed a HFD treated with vehicle. Taken together, these results suggest that mice fed a HFD and treated with ibrutinib have improved glycaemic regulation.

TABLE 1 Basic metabolic parameters measured in interventional study

**TABLE 1:** Basic metabolic parameters measured in interventional study

	Chow	Chow + ibrutinib (30 mg·kg^−1^)	HFD + Veh	HFD + ibrutinib (3 mg·kg^−1^)	HFD + ibrutinib (30 mg·kg^−1^)
Body weight (g)	31.24 ± 0.43*	30.65 ± 0.51*	36.1 ± 0.80	33.55 ± 0.79	31.21 ± 0.31
Weight gain from baseline (g)	7.03 ± 0.54*	6.84 ± 1.85*	11.22 ± 0.51	9.42 ± 0.508	9.91 ± 0.83
Kidney weight (g)	0.3088 ± 0.007	0.3362 ± 0.002	0.3295 ± 0.011	0.3198 ± 0.010	0.3029 ± 0.007
Liver weight (g)	1.185 ± 0.038*	1.187 ± 0.065*	2.817 ± 0.048	1.256 ± 0.048*	1.287 ± 0.0442*
Inguinal fat weight (g)	0.394 ± 0.043*	0.3837 ± 0.003*	0.4531 ± 0.037	0.3505 ± 0.089*	0.3993 ± 0.028*
ALT (U·L^−1^)	45.40 ± 19.95	46.00 ± 11.68	61.04 ± 13.16	58.47 ± 9.031	54.63 ± 7.16
AST (U·L^−1^)	80.88 ± 5.887*	92.01 ± 22.67	120.4 ± 16.55	115 ± 11.87	119 ± 12.18

Body weight, body weight gain from baseline, inguinal fat weight, kidney weight, liver weight, inguinal fat weight, ALT, and AST were measured in mice fed a standard diet (chow) or a high‐fat diet (HFD) for 12 weeks. HFD mice were treated with either vehicle or ibrutinib (3 or 30 mg·kg^−1^ p.o.) five times per weeks between weeks 7 and 12. Data are expressed as mean ± SEM from 9/10 mice per group.

* *P* < 0.05, significantly different from HFD + Veh. one‐way ANOVA with Bonferroni post hoc test.

Having demonstrated that treatment with ibrutinib improved functional parameters of glycaemic regulation in HFD fed mice, we next investigated the effects of ibrutinib treatment on the insulin signalling pathway in the liver. When compared to mice fed a chow diet, mice fed a HFD treated with vehicle have an increase in the phosphorylation on Ser^307^ of IRS‐1 (Figure 1e,f), resulting in a reduction in phosphorylation of downstream mediators Akt on Ser^473^ and GSK‐3β on Ser^9^ (Figure 1e,f). Mice fed a HFD and treated with ibrutinib demonstrated decreased phosphorylation of Ser^307^ of IRS‐1, attenuating the decrease in phosphorylation of Akt and GSK‐3β, compared with mice fed a HFD (Figure 1e,f). Thus, the observed improvement in glycaemic regulation in ibrutinib treated HFD‐fed mice resulted from preserved insulin signalling through IRS‐1/Akt/GSK‐3β.

HFD feeding to mice is associated with the development of hepatosteatosis in mice. We therefore quantified fat deposition in the liver using Oil Red O staining. When compared to chow diet, mice fed a HFD and treated with vehicle had a significant increase in Oil Red O staining in the liver (Figure 1g,h). Treatment of HFD fed mice with ibrutinib (3 or 30 mg·kg^−1^) resulted in a significant reduction in Oil Red O staining in the liver (Figure 1g,h), demonstrating less lipid deposition in the liver and hence reduced hepatic steatosis compared to vehicle treated controls. We next wanted to investigate the expression of genes involved in hepatic lipid processing. Mice fed a HFD had increased expression of genes that participate in free fatty acid uptake (CD36, SCREB, UCP2, and FASN) as well as genes involved in beta oxidation of free fatty acids (CPT, LCAD, and SCD1) (Figure 1i). Treatment with ibrutinib attenuated the increase in expression of gene associated with FFA uptake and beta oxidation (Figure 1i,j) and could account in part for the reduced lipid accumulation seen in mice fed a HFD and treated with ibrutinib.

**FIGURE 2:**
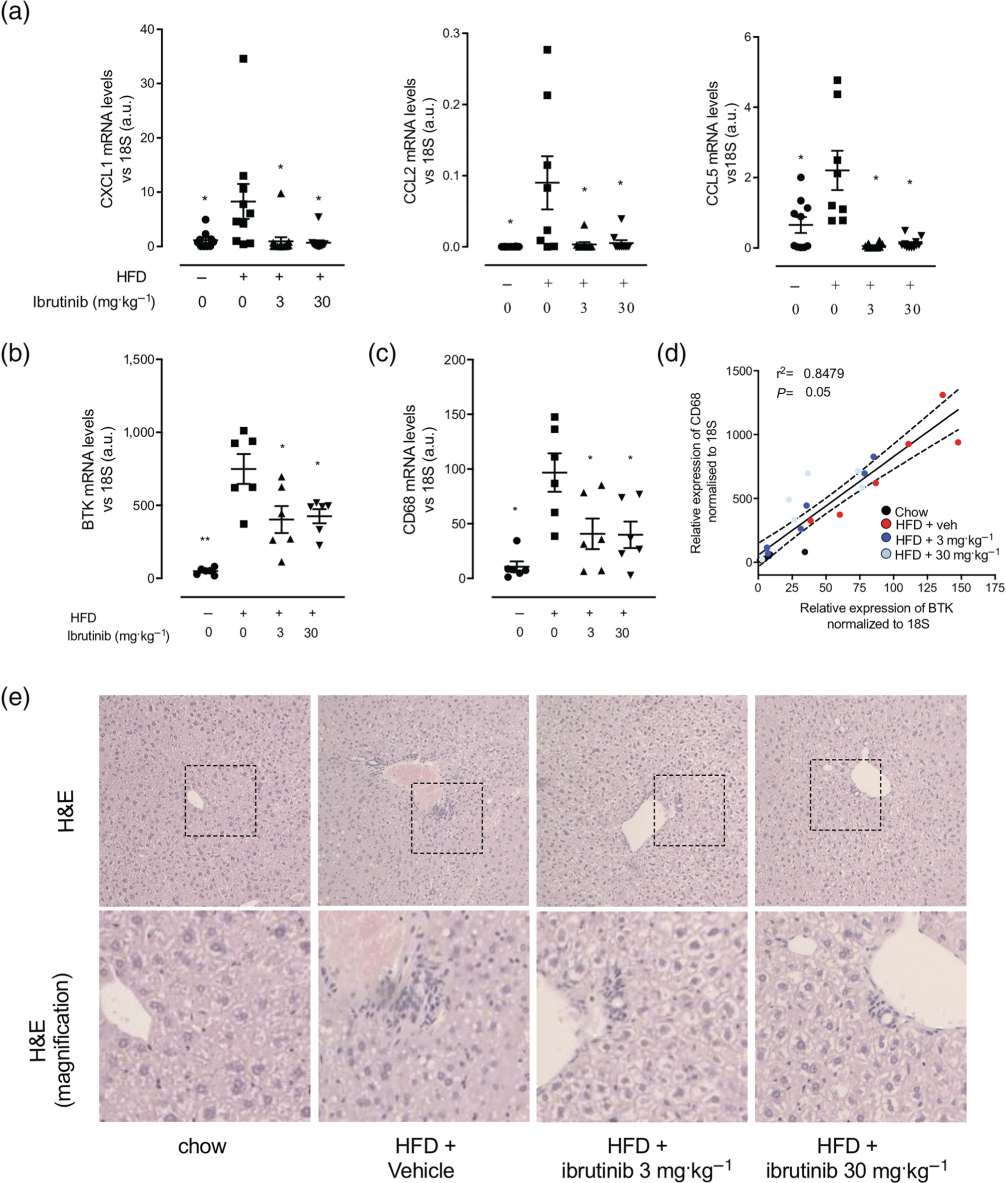
Ibrutinib treatment reduces inflammatory cell recruitment in the liver of mice fed a HFD. (a) Relative gene expression of *CXCL1*, *CCL2*, and *CCL5* were assessed by qPCR and normalized to *18S*. Relative gene expression of *CD68* (b) and *BTK* (c) were assessed by qPCR and normalized to *18S* in the liver. (d) Correlation of relative gene expression of *CD68* to *BTK* in the liver. Correlation was calculated by linear regression including 95% confidence intervals. (e) Immune cell infiltration was assessed by H&E staining. Inset images are 4× digital zoom. Data shown are individual values with means ± SEM. ^*^ *P* < 0.05, significantly different from HFD + Veh; one‐way ANOVA with Bonferroni post hoc test

A critical step in the initiation of hepatic lipid uptake and in the development of diabetes is the accumulation and activation of immune cells within tissues including the liver. Mice fed a HFD have increased levels of chemokines (CXCL1, CCL2, and CCL5) mRNA expression in liver tissue (Figure 2a), which was coupled with increased immune cell infiltration. Ibrutinib treatment to mice fed a HFD resulted in reduced monocyte/macrophage chemokine expression (Figure 2a) and reduced immune cell infiltration (Figure 2e). In the liver, we observed an increased expression of CD68 (a gene marker predominantly expressed in macrophages) in mice fed a HFD (Figure 2b) directly correlated with a significant increase in expression of BTK (Figure 2c,d). Treatment of HFD fed mice with ibrutinib (3 or 30 mg·kg^−1^) resulted in a significant decrease in expression of CD68 and BTK in the liver (Figure 2b–d) suggesting less infiltration of inflammatory monocytes/macrophages.

### 3.2 Inhibition of BTK with ibrutinib reduces inflammation in the liver of HFD fed mice

**FIGURE 3:**
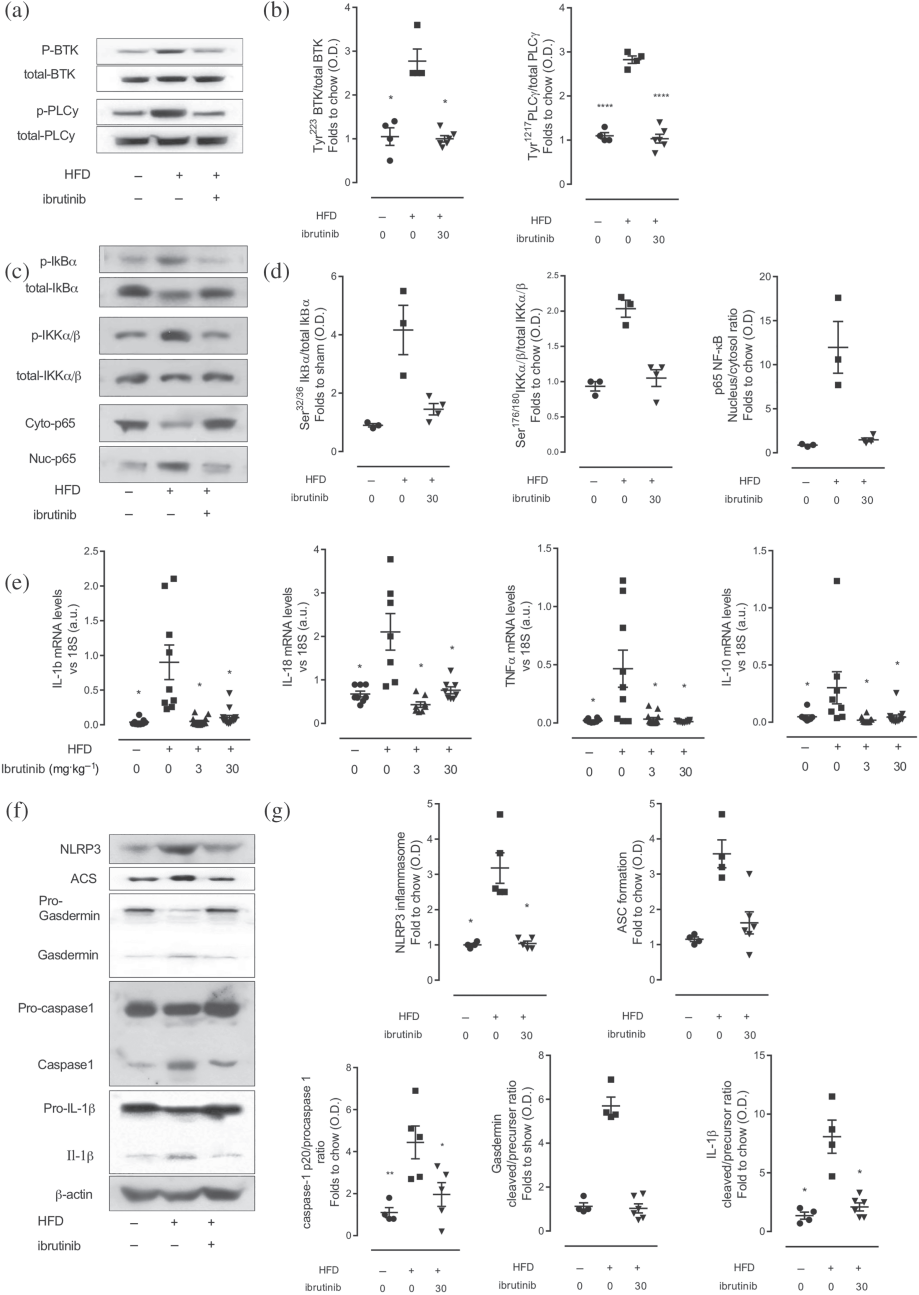
Ibrutinib treatment reduces inflammation in the liver of mice fed a via inhibition of NF‐κB and the NLRP3 inflammasome. (a) Representative western blots for phosphorylation of Tyr^223^ on BTK in the liver and normalized to total BTK; for phosphorylation of Tyr^1217^ on PLCγ in the liver and normalized to total PLCγ and (b) quantified using densitometry. (c) Representative western blots for phosphorylation of Ser^32/36^ on IKBα in the liver and normalized to total IKBα; for phosphorylation of Ser^176/180^ on IKKα in the liver and normalized to total IKKα; (d) nuclear translocation of p65 and (e) quantified using densitometry. Data shown are individual values with means ± SEM; *n* = 3–6 per group. ^*^ *P* < 0.05, significantly different from HFD + Veh; one‐way ANOVA with Bonferroni post hoc test. (f) Relative gene expression of *IL‐1β*, *IL‐18*, *TNFα*, and *IL‐10* were assessed by qPCR and normalized to *18S*. **(**g) Representative western blots for NLRP3 inflammasome assembly, ASC, formation of gasdermin, the proteolytic cleavage of pro‐caspase 1 to caspase 1 and formation of IL‐1β normalized to β‐actin and (h) quantified using densitometry. Data shown are individual values with means ± SEM; *n* = 6–10 per group. ^*^ *P* < 0.05, significantly different from HFD + Veh; one‐way ANOVA with Bonferroni post hoc test

We next wanted to investigate if the improvements in the diabetic phenotype seen in mice treated with ibrutinib could be attributed to reduced activation of pro‐inflammatory pathways in the liver. BTK has been implicated in pro‐inflammatory signalling; therefore, inhibiting BTK in infiltrating monocytes/macrophages could protect against the development of insulin resistance, through reduced production of soluble inflammatory mediators. When compared to mice fed chow diet, liver tissue from mice fed a HFD exhibited a significant increase in the phosphorylation of Tyr^223^ and hence activation of BTK (Figure 3a,b), resulting in increased downstream signalling through PLCγ (Figure 3a,b). Mice fed a HFD treated with ibrutinib have significantly reduced activation of BTK and downstream effector PLCγ (Figure 3a,b).

Two of the key pathways involved in the initiation and progression of metabolic inflammation in the liver are the NF‐κB pathway and the NLRP3 inflammasome. Therefore, we investigated whether inhibition of BTK with ibrutinib would result in reduced activation of these two critical signalling pathways in the liver. Firstly, we examined NF‐κB signalling. When compared to mice fed chow diet, liver tissue from mice fed a HFD exhibited a significant increase in the phosphorylation of Ser^32/36^ in IκBα and, hence, activation (phosphorylation) of the IKK complex (Figure 3c,e), allowing for increased nuclear translocation of the NF‐κB subunit p65 to the nucleus where its acts a nuclear transcription factor (Figure 3d,e). Indeed, we observed increased cytokine mRNA expression (IL‐1β, IL‐18, TNFα, and IL‐10) (Figure 3f). Liver tissues from mice fed a HFD and treated with ibrutinib showed a significant reduction in the degree of phosphorylation of IκBα on Ser^32/36^ and, hence, reduced activation of the IKK complex (Figure 3c,e) and as a consequence reduced nuclear translocation of the p65 NF‐κB subunit to the nucleus (Figure 3d,e). As a result, ibrutinib treatment of mice fed a HFD attenuated the increase in the gene expression of IL‐1β, IL‐18, TNFα, and IL‐10 in the liver (Figure 3f).

Secondly, we examined NLRP3 function in liver tissues. Mice fed a HFD demonstrated assembly of the NLRP3 inflammasome in the liver, allowing for the sequential proteolytic cleavage of gasdermin D, and caspase‐1, ultimately producing mature IL‐1β (Figure 3g,h). Mice fed a HFD and treated with ibrutinib have significantly reduced formation of the NLRP3 inflammasome and, hence, reduced proteolytic cleavage of pro‐gasdermin D and pro‐caspase 1 in the liver resulting in attenuated formation of active IL‐1β (Figure 3g,h). Collectively, these results demonstrate that chronic treatment with ibrutinib inhibits BTK activity and reduces NF‐κB activation and formation of the NLRP3 inflammasome in the liver, thus reducing hepatic pro‐inflammatory gene expression and cytokine production. Reducing hepatic inflammation, in part, protects against the development of peripheral insulin resistance in mice fed a HFD (Saltiel & Olefsky, 2017; de Luca & Olefsky, 2008).

### 3.3 Treatment with ibrutinib reduces inflammation in adipose tissue of HFD fed mice

**FIGURE 4:**
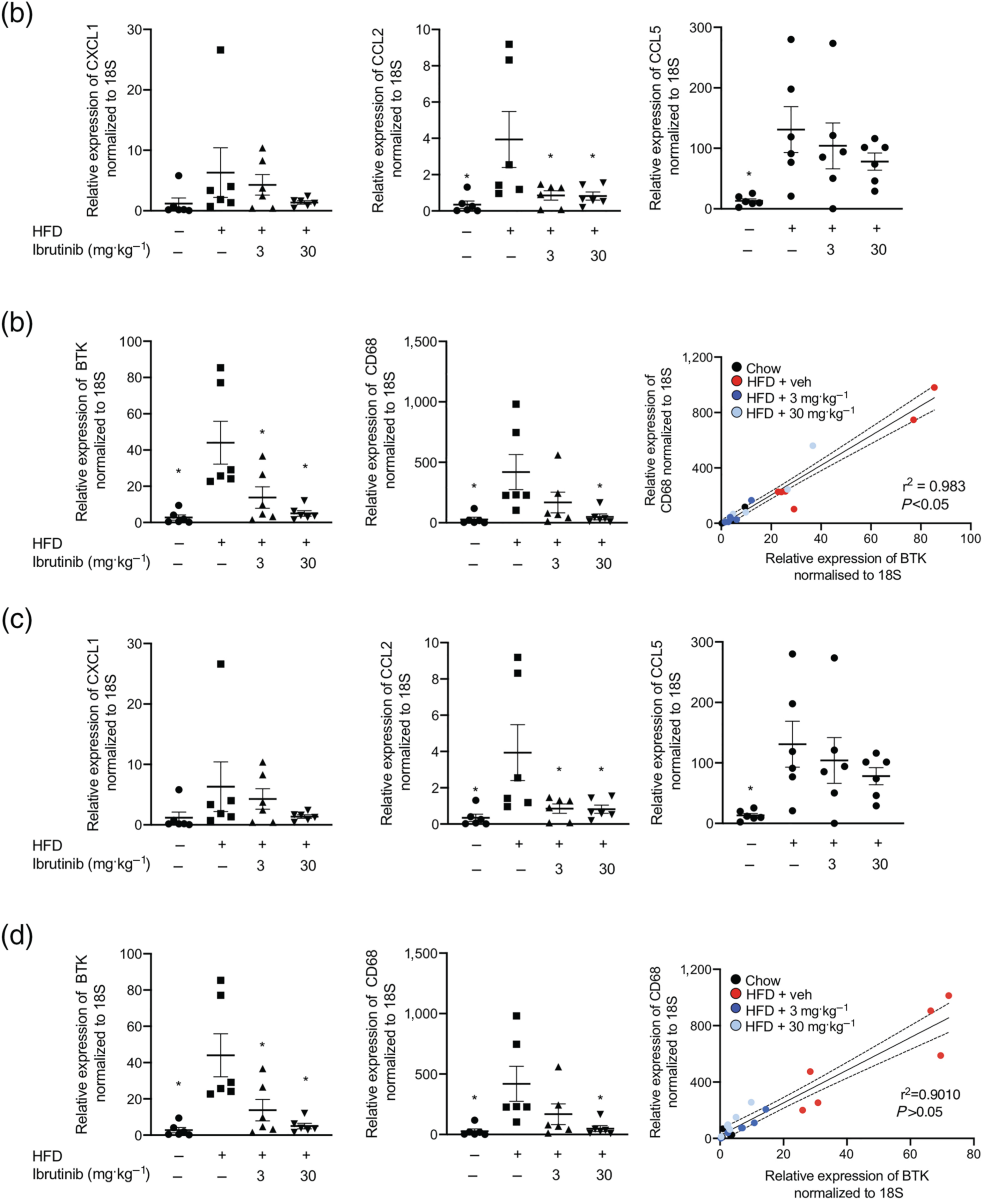
Ibrutinib treatment reduces inflammation in adipose tissue of mice fed a HFD. Relative gene expression of *CXCL1*, *CCL2*, and *CCL5* were assessed by qPCR and normalized to *18S* in epididymal (a) and inguinal (c) adipose tissues. Relative gene expression of *CD68* and *BTK* were assessed by qPCR and normalized to *18S* in the liver. Data shown are individual values with means ± SEM. *n* = 6 per group. ^*^ *P* < 0.05, significantly different from HFD + Veh; one‐way ANOVA with Bonferroni post hoc test. Correlation of relative gene expression of *CD68* to *BTK* in epididymal (b) and inguinal (d) adipose tissues. Correlation of BTK and CD68 mRNA levels. Correlation was calculated by linear regression including 95% confidence intervals

Following HFD feeding, there is a significant expansion of peripheral adipose tissue deposits that is typically associated with an increase in the adipose monocyte/macrophage population. Mice fed a HFD had significantly larger fat pads compared to chow fed mice as expected (Table 1). Treatment with ibrutinib attenuated the increases in weight of inguinal and epididymal fat pads compared to vehicle treated control (Table 1). We also show that mice fed a HFD have high expression of monocyte/macrophage chemoattractants (CXCL1, CCL2, and CCL5) in the epididymal fat and in the inguinal fat pads (Figure 4a,c), which was attenuated by ibrutinib treatment (Figure 4a,c). Within the adipose tissue, there was an increased expression of CD68 in mice fed a HFD (Figure 4b,d), and this positively correlated with a significant increase in expression of BTK (Figure 4b,d), indicative of increased macrophage content. Treatment of HFD mice with ibrutinib (3 or 30 mg·kg^−1^) resulted in significantly decreased expression of CD68 and BTK in the adipose tissue (Figure 4b,d) suggesting less infiltration of inflammatory monocytes/macrophages.

### 3.4 Treatment with ibrutinib reduces inflammation in the kidney of HFD fed mice

**FIGURE 5:**
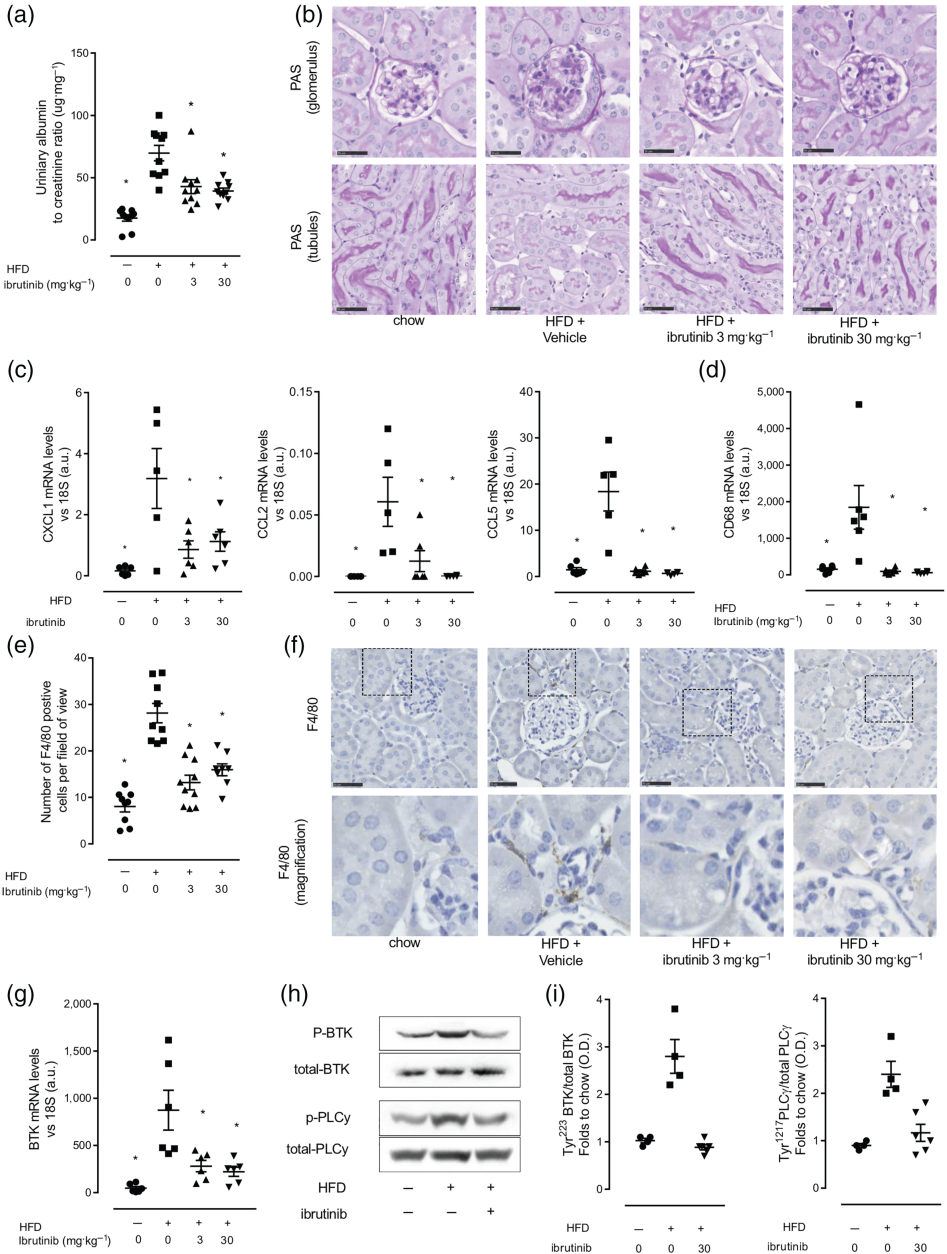
Ibrutinib treatment prevents the development proteinuria in mice fed a HFD. (a) Urinary albumin to creatinine ratio (ACR). (b) Kidney sections were stained with periodic acid Schiff staining to assess histological changes. in vivo data (*n* = 9–10 per group) (c) Relative gene expression of *CXCL1*, *CCL2*, *CCL5*, and *CD68* was assessed by qPCR and normalized to *18S*. Data shown are individual values with means ± SEM; *n* = 5–6 per group. ^*^ *P* < 0.05, significantly different from HFD + Veh; one‐way ANOVA with Bonferroni post hoc test. (f) Macrophage infiltration was assessed by immunohistochemical staining for macrophage marker F4/80 and quantified (e); scale bars measure 50 μm. Inset images are 4× digital zoom. Data shown are individual values with means ± SEM; *n* = 8–10 per group. ^*^ *P* < 0.05, significantly different from HFD + Veh; one‐way ANOVA with Bonferroni post hoc test. (g) Relative gene expression of *BTK* was assessed by qPCR and normalized to *18S*. Data shown are individual values with means ± SEM; *n* = 5–6 per group. ^*^ *P* < 0.05, significantly different from HFD + Veh; one‐way ANOVA with Bonferroni post hoc test. (h) Representative western blots for phosphorylation of Tyr^223^ on BTK in the kidney and normalized to total BTK; for phosphorylation of Tyr^1217^ on PLCγ in the kidney and normalized to total PLCγ and (i) quantified using densitometry. Data shown are individual values with means ± SEM; *n* = 3–6 per group. ^*^ *P* < 0.05, significantly different from HFD + Veh; one‐way ANOVA with Bonferroni post hoc test

Metabolic inflammation coupled with hyperglycaemia leads to the development of microvascular complications including diabetic nephropathy. Mice fed a HFD (treated with vehicle) had an increased urinary albumin to creatinine ratio (ACR), compared to mice fed a chow diet (Figure 5a). This is a key pathophysiological read out for proteinuria and a hallmark of early stage diabetic nephropathy (Figure 5a). This was accompanied by classical histological changes in the kidney that are consistent with the development of microvascular disease i.e. glomerular hypertrophy, thickening of the basement membrane and loss of brush borders at the level of the S1–S2 segment of the proximal convoluted tubules (Figure 5b). Prolonged treatment of HFD fed mice with ibrutinib (3 or 30 mg·kg^−1^) attenuated both the proteinuria and the associated histological signs of glomerular and tubular degeneration (Figure 5a,b). Infiltration of inflammatory macrophages is a common finding in diabetic nephropathy. We show that mice fed a HFD have high expression of the monocyte/macrophage chemoattractants (CXCL1, CCL2, and CCL5) (Figure 5c) and an increased number of F4/80^+^ macrophages in the kidney which was attenuated by ibrutinib treatment (3 or 30 mg·kg^−1^) (Figure 5e,f). Additionally, increased expression of macrophage marker CD68 (Figure 5d) was associated with an increase in expression of BTK, suggesting the primary source of increased BTK is from infiltrating monocytes and macrophages (Figure 5g). Kidney tissues from mice fed a HFD exhibited a significant increase in the phosphorylation of Tyr^223^ on BTK, suggesting increased activation of BTK, resulting in an increase downstream tyrosine phosphorylation of PLCγ demonstrating that BTK is activated and signalling in the diabetic kidney (Figure 5h,i). Mice fed a HFD and treated with ibrutinib have significantly reduced activation of BTK and downstream effector PLCγ. Taken together, these results suggest that increased macrophage accumulation in the kidney could be the source of increased BTK activation seen in kidney of mice fed a HFD.

Activation of both NF‐κB and the NLPR3 inflammasome have been associated with macrophage accumulation and progression of diabetic nephropathy. Critically, ibrutinib treatment attenuated the activation of both the NF‐κB (Figure S2A, B) and NLRP3 inflammasome activity (Figure S2C, D) in the kidney of mice fed a HFD. Our data demonstrate that oral dosing with ibrutinib inhibits BTK signalling protecting against the development of proteinuria by reducing immune cell recruitment and inhibiting pro‐inflammatory gene expression and cytokine production through its effects on NF‐κB and the NLRP3 inflammasome.

### 3.5 Ibrutinib treatment reduces NF‐κB and NLRP3 inflammasome activation in murine and human macrophages

**FIGURE 6:**
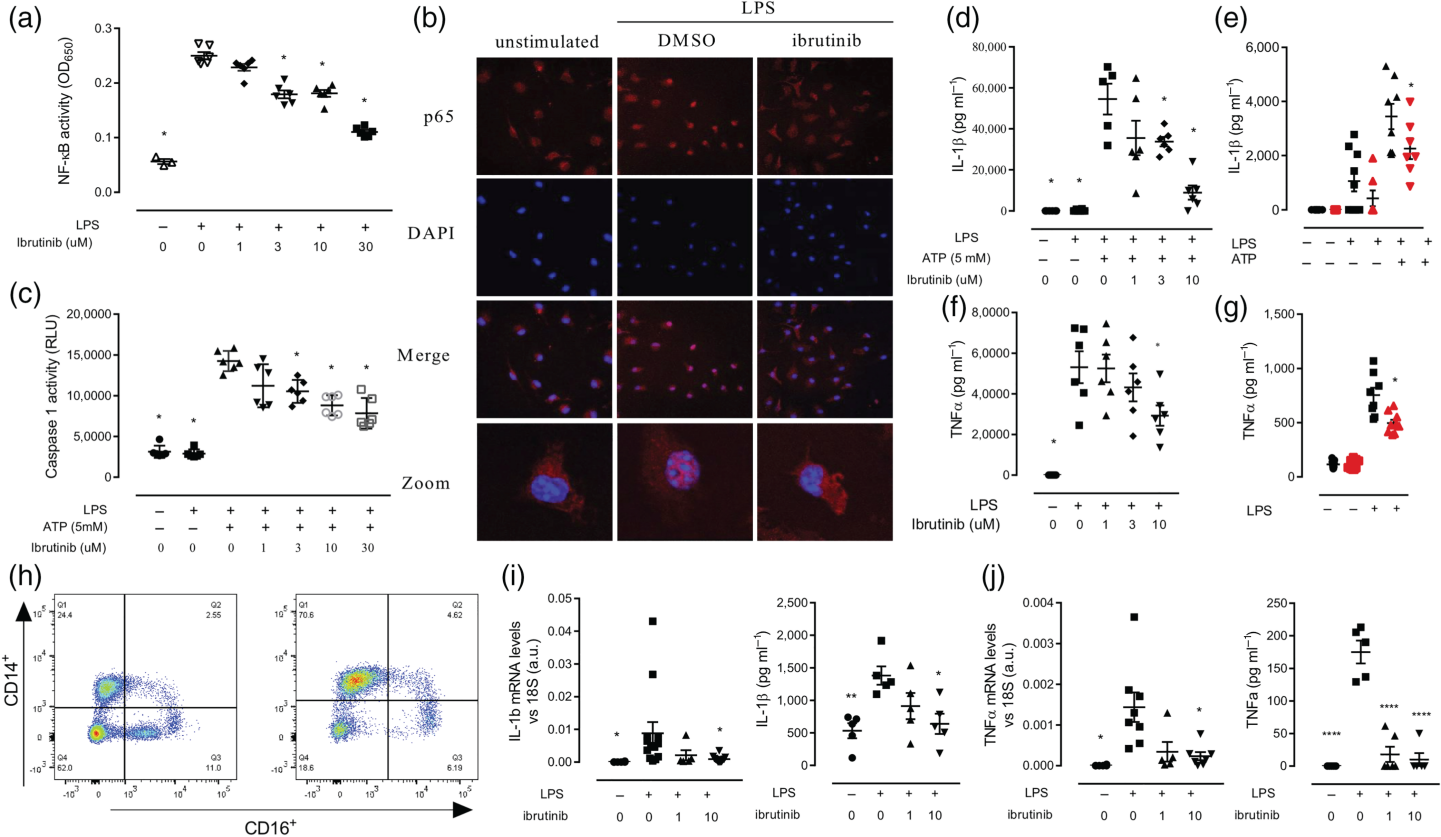
Ibrutinib treatment reduces NF‐κB and NLRP3 activation in macrophages. (a) NF‐κB and AP1 activity assay in RAW Blue cells. Data shown are individual values with means ± SEM; *n* = 4 independent experiments. (b) Bone marrow derived macrophages (BMDMs) pretreated with ibrutinib (10 μM) prior to LPS (0.1 μg·ml^−1^) stimulation 30 min followed by p65 staining. Confocal microscopy images are illustrative of two separate experiments; all images were captured at magnification 60×. (c) Caspase 1 activity was measured using luminescence. Data shown are individual values with means ± SEM; *n* = 4 independent BMDM preparations. (d, f) Cytokine (IL‐1β and TNFα) secretion measured by ELISA in WT BMDM. Summary data of *n* = 6 independent BMDM preparations. (e, g) Cytokine (IL‐1β and TNFα) secretion measured by ELISA in BMDM from WT and XID. Data shown are individual values with means ± SEM; *n* = 6 independent BMDM preparations. (h) Human monocytes are isolated from leukocyte cones and purified by magnetic bead purification. Representative flow cytometry plots showing monocyte enrichment; monocytes are defined as CD14^+^/CD16. (i) Cytokine secretion (IL‐1β and TNFα) was assessed by Bead based ELISA kit and relative gene expression of *IL‐1β* and *TNFα* were assessed by qPCR and normalized to 18S. ^*^ *P* < 0.05, significantly different from HFD + Veh; one‐way ANOVA with Bonferroni post hoc test

Macrophages are hypothesized to be a key cell type involved in the local amplification of metabolic inflammation in peripheral tissues following HFD feeding in this study. Therefore, we wanted to directly confirm that ibrutinib inhibits the activation of both NF‐κB and the NLRP3 inflammasome in macrophages. To investigate this, we first used a macrophage cell line stably transfected with a secreted alkaline phosphatase reporter gene under the transcription regulation of NF‐κB. Macrophages stimulated with LPS for 6 h exhibited a strong induction of NF‐κB activity (Figure 6a) which could be inhibited when macrophages were pre‐incubated with ibrutinib (1–30 μM) to inhibit BTK 60 min before LPS stimulation (Figure 6a). No cytotoxicity was observed in macrophages treated with up to 30 μM of ibrutinib for 9 h in an MTT assay (Figure S3A). We then confirmed that BTK inhibition with ibrutinib resulted in less NF‐κB activation in primary murine bone marrow derived macrophages (BMDM) (Figure S3B). LPS stimulation induced translocation of p65 from the cytoplasm to the nucleus in BMDM, which was inhibited by pretreatment with ibrutinib (10 μM; for 1 h prior to LPS stimulation; Figure 6b). Stimulation of BMDMs with LPS also resulted in the up‐regulation of pro‐inflammatory gene expression including IL‐1β, IL‐18, TNFα, and IL‐6, which was reduced by pre‐treating with ibrutinib, in a dose‐dependent manner (Figure S3C).

Next, we demonstrated BTK inhibition with ibrutinib blocked the formation of the NLRP3 inflammasome in primary murine BMDM using a caspase‐1 activity assay. Incubating BMDMs with LPS alone does not result in activation of caspase‐1 (Figure 6c,) as a second signal is required. To provide this second stimulus, ATP (5 mM) was added to BMDM in the last 60 min of LPS challenge, which resulted in a significant increase in caspase‐1 activity (Figure 6c). Pretreatment of BMDM with ibrutinib (1–30 μM) 60 min prior to LPS + ATP stimulation resulted in a dose‐dependent inhibition of caspase‐1 activity (Figure 6c).

The downstream effects of reduced NF‐κB (TNFα) and NLRP3 inflammasome (IL‐1β) on pro‐inflammatory cytokine secretion by BMDM were also confirmed. BMDM were stimulated with LPS for 8 h and supernatants collected and cytokine levels measured by ELISA; for IL‐1β secretion, ATP was added in the last 60 min. Pretreatment with ibrutinib 60 min prior to LPS stimulation significantly inhibited the secretion of pro‐inflammatory cytokines IL‐1β and TNFα in a dose‐dependent manner (Figure 6d,f) by macrophages. To confirm that the effect of ibrutinib was BTK specific, BMDM from WT and XID mice (which have an inactive BTK) were treated with LPS to measure TNFα production and LPS + ATP to measure IL‐1β production. Importantly, we demonstrate that TNFα and IL‐1β production from XID mice BMDM was significantly reduced (Figure 6e,g). These experiments demonstrate a clear role for BTK in regulating both NF‐κB and NLPR3 inflammasome‐dependent cytokine production and secretion in macrophages.

To test the translational relevance of our findings obtained in murine macrophages, we studied the activation of pro‐inflammatory gene expression and the secretion of pro‐inflammatory cytokines in human monocyte derived macrophages (hMoDM). Human monocytes were isolated from leukocyte cones obtained from healthy volunteers and enriched by column separation (Figure 6h) and then differentiated into macrophages. On Day 7 of differentiation, hMoDM were pretreated with LPS to induce inflammatory gene expression. Pretreatment with ibrutinib (10 μM) 60 min prior to LPS stimulation inhibited the increase in pro‐inflammatory gene expression (IL‐1β and TNFα) and cytokine secretion seen in hMoDM treated with LPS alone (Figure 5i,j). Pretreatment with ibrutinib prior to LPS stimulation significantly inhibited the secretion of both NF‐κB and NLRP3 dependent cytokines TNFα and IL‐1β in a dose dependant manner (Figure 6i,j). These experiments clearly demonstrated that inhibition of BTK with ibrutinib had potent anti‐inflammatory effects in human macrophages, resulting from the inhibition of both NF‐κB and NLRP3 inflammasome activity.

## 4 DISCUSSION

In this study, we showed for the first time that BTK was activated as a result of HFD feeding in a model of diet‐induced metabolic inflammation. BTK has previously been shown to regulate pro‐inflammatory pathways including NF‐κB and the NLRP3 inflammasome. We, therefore, set out to investigate if the anti‐cancer agent ibrutinib could represent a novel drug repositioning opportunity for the treatment of HFD induced metabolic inflammation. The main findings of this preclinical study are that chronic treatment of mice fed a HFD with ibrutinib inhibits the activation and downstream signalling of BTK and inhibits the activation of NF‐κB and the NLRP3 inflammasome in the liver and kidney. Reduced metabolic inflammation in the liver and adipose tissue resulted in improved glycaemic control via restoration of IRS‐1/Akt/GSK‐3β signalling pathway in the liver. These improvements in glycaemic control were independent of changes in body weight or calorific intake. Importantly, treatment with ibrutinib also protected mice from the development of hepatosteatosis and proteinuria. We demonstrated that the expression of BTK significantly correlated with expression of CD68 a marker of infiltrating monocytes/macrophages, while ibrutinib treatment significantly reduced the expression of monocyte/macrophage chemoattractants in the liver, adipose, and kidney. We also showed, in primary murine and human macrophages, that ibrutinib treatment reduced LPS‐stimulated NF‐κB activation and attenuated caspase‐1 activity, resulting in a reduction in pro‐inflammatory gene expression and a reduction in secreted cytokines (IL‐1β and TNFα). Critically, our work has identified a novel therapeutic arget in diet induced inflammation, namely, monocyte/macrophage expressed BTK. Importantly, we provide “proof‐of‐concept” data that ibrutinib reduces HFD‐induced inflammation and could be a candidate for repurposing to treat inflammation in metabolic diseases.

Identifying that monocytes/macrophages are another leukocyte where BTK is highly expressed broadens interest in this molecule both in metabolic inflammation and potentially in other inflammatory diseases. BTK expression was positively correlated with expression of CD68 a monocyte/macrophage marker in mice fed a HFD, suggesting that the increase in BTK seen in the diabetic liver, adipose tissue, and kidney is from infiltrating monocytes/macrophages. We suggest that systemic ibrutinib treatment in vivo inhibited cellular BTK activation in macrophages of the liver and kidney. The activation of BTK and the subsequent signalling cascade starts with the phosphorylation of BTK at Tyr^223^ which then phosphorylates PLCγ at Tyr^1217^ (O'Riordan et al., 2019). In our model, mice fed a HFD exhibited increased phosphorylation of BTK in the liver and kidney and that this activation was inhibited with ibrutinib treatment. We hypothesized that BTK/PLCγ signalling may regulate both the NF‐κB signalling and the activation of the NLRP3 inflammasome. Previous studies have shown a mechanistic link between PLCγ activation and NF‐κB activity in B‐cells (Moscat, Diaz‐Meco, & Rennert, 2003), but no such link had been described in cells of the myeloid lineage. In our model, we showed that, in primary murine macrophages, BTK regulated both NF‐κB and NLRP3 inflammasome activity, possibly through PLCγ.

Myeloid cell activation in the liver and adipose tissue has been consistently shown to be the key driver of metabolic inflammation resulting in increased activation of numerous pathways including NF‐κB. Interventional studies have reported the beneficial effects of NF‐κB inhibition on the development of insulin resistance (Chiazza et al., 2015; Zhu, Han, Yuan, Xue, & Pang, 2018; Benzler et al., 2015). Mice with myeloid specific deletion of IKK‐β fed a HFD do not develop insulin resistance, while mice fed a HFD with hepatocyte specific deletion of IKK develop less severe insulin resistance (Arkan et al., 2005; Ke et al., 2015). However, deletion of NF‐κB activity in skeletal muscle does not protect mice from the development of insulin resistance (Röhl et al., 2004). Taken together these published studies demonstrate that while systemically inhibiting the NF‐κB pathways does prevent the development of insulin resistance, it is the myeloid specific inhibition that is the critical cellular target in vivo. We would postulate that the BTK expressed in monocytes/macrophages within metabolic tissues (liver, adipose tissue and kidney) is the pharmacological target of ibrutinib, because BTK expression in monocytes/macrophages is significantly higher than in hepatocytes or adipocytes. Myeloid activation of NF‐κB is correlated with functional decline in models of HFD induced obesity and insulin resistance. Indeed, here we show that ibrutinib treatment of mice fed a HFD results in reduced activation of the NF‐κB pathway in both the kidney and liver, which as a consequence of HFD feeding have increased accumulation of macrophages. Activation of NF‐κB results in the production of key components of the NLPR3 inflammasome pathway including pro‐caspase 1 and pro‐IL‐1β. Ablation of the NLRP3 inflammasome has been shown to improve glycaemic control in obese mice by reducing caspase‐1 cleavage and Il‐1β/IL‐18 activation (Vandanmagsar et al., 2011). Here, we have demonstrated that treatment with ibrutinib of mice fed a HFD attenuated the formation of the NLRP3 complex and blocked the proteolytic cleavage of pro‐caspase 1 to active caspase 1 resulting in attenuated production of IL‐1β in the liver and kidney.

One important consequence of having reduced metabolic inflammation is improved glycaemic control. We demonstrate that ibrutinib treatment of mice fed a HFD improved glycaemic control (measured as OGTT) and lowered both terminal blood glucose and insulin levels. Mechanistically, we also show that treatment with ibrutinib of mice fed a HFD decreased the phosphorylation of Ser^307^ on IRS‐1 and increased activation of Akt and GSK‐3β in the liver. It should be noted that although phosphorylation of Ser^307^ is a widely used marker of insulin resistance, it is not causative in vivo (Copps et al., 2010). Some studies have reported that IKK‐β can also regulate insulin sensitivity through direct phosphorylation of IRS‐1 suggesting that there may also be a transcription‐independent mechanism of the NF‐κB pathways in our model (Arkan et al., 2005). Indeed, we show that mice fed a HFD and treated with ibrutinib have less activation of IKK‐β, which correlates with a reduction in IRS‐1 phosphorylation. Thus, we have demonstrated for the first time a mechanistic link between BTK inhibition and IKK‐β/IRS‐1 signalling in vivo.

Even with careful management of blood glucose and strategies to lower circulating lipids, microvascular complications develop over time in patients with T2D. These complications occur predominantly in tissues where glucose uptake is insulin‐independent, such as the kidney, retina and vascular endothelium, as these tissues are exposed to glucose levels close to blood glucose levels. The extent of inflammatory cell accumulation in the diabetic kidney has been associated with the decline of renal function, suggesting a causative link (Macisaac, Ekinci, & Jerums, 2014). We speculated that BTK is a pivotal kinase in tissue resident macrophages needed for the production of chemokines that recruit immune cells to sites of inflammation. Tissue levels of key chemokines were reduced in the liver, adipose tissue and kidney in mice fed a HFD and treated with ibrutinib, including the chemokine CCL2 which is pivotal for monocyte/macrophage recruitment. In many preclinical models of diet induced obesity and microvascular disease genetic deletion or pharmacological inhibition of CCR2 dramatically reduces macrophage recruitment and protects the kidney from functional decline (Awad et al., 2011; Sayyed et al., 2011). In the present study, ibrutinib treatment significantly reduced macrophage accumulation due to lower expression of CCL2 mRNA in the kidney of mice fed a HFD. These results are consistent with previous data generated in experimental models of diabetic nephropathy, where macrophage accumulation is reduced in *Ccl2* ^*−/−*^ mice. Here, inhibition of BTK with ibrutinib reduced both NF‐κB and NLRP3 inflammasome activation in the kidney, reduced classical histological markers of early diabetic nephropathy, and protected mice from the development of proteinuria. These data are consistent with other studies reporting that targeting the inflammatory component of early stage diabetic kidney disease improves functional outcome (Anders, 2016). Indeed, individually targeting NF‐κB and the NLRP3 inflammasome have been shown to be efficacious in protecting against microvascular disease in diabetes. While this accumulation of evidence from genetic modifications in preclinical models validated this pathway as a therapeutic target, our study is unique in identifying an upstream molecular target and demonstrating the efficacy of our approach using an orally available, drug‐repurposing candidate. Using an FDA approved medicine, ibrutinib, we decreased NF‐κB and NLRP3 inflammasome driven inflammation, improved glycaemic control and elicited protection from microvascular damage in this model of diet‐induced metabolic inflammation.

In conclusion, the concept that the development of T2D is intrinsically linked to the extent of myeloid cell activation is emerging in the field of diabetic medicine. Here, we demonstrate that BTK expression and activation are increased in mice fed a HFD, and this significantly correlated with monocyte/macrophage infiltration into the liver and kidney. Therapeutic treatment with the FDA approved BTK inhibitor ibrutinib not only reduced the extent of monocyte/macrophage infiltration into the liver and kidney of mice fed a HFD but also attenuated the activation of NF‐κB and the NLRP3 inflammasome. Critically, we also demonstrated that treatment with ibrutinib reduced systemic inflammation and resulted in improved glycaemic regulation, lowering blood glucose and insulin levels, by restoring signalling through IRS‐1/Akt/GSK‐3β. Improved glycaemic control also protected mice fed a HFD from the development of hepatosteatosis and proteinuria. Taken together, we have identified an FDA approved medication that has many of the ideal properties of a candidate medication for repurposing into the treatment of metabolic inflammation, in diseases such as Type 2 diabetes.

## AUTHOR CONTRIBUTIONS

G.S.D.P., M.C., H.M.A.T., F.C., D.C., R.V., C.E.O., and L.Z. performed the experiments. G.S.D.P., M.C., and H.M.A.T. analysed the results and made the figures. G.S.D.P., P.B., N.G., D.R.G., and C.T. designed the research. G.S.D.P, D.R.G., and C.T. wrote the paper. M.M.Y. contributed to the discussion. All authors provided critical revision of the manuscript.

## CONFLICT OF INTEREST

The authors declare no conflicts of interest.

## DECLARATION OF TRANSPARENCY AND SCIENTIFIC RIGOUR

This Declaration acknowledges that this paper adheres to the principles for transparent reporting and scientific rigour of preclinical research as stated in the BJP guidelines for Design & Analysis, Immunoblotting and Immunochemistry and Animal Experimentation, and as recommended by funding agencies, publishers and other organisations engaged with supporting research.

## Supporting information


**Figure S1:** A) C57BL/6 mice, fed a standard diet (chow) or a high‐fat diet (HFD) for 12 weeks and OGTT was performed at week 6 and week 12 and quantified B). C57BL/6 mice, fed a standard diet (chow) were treated with vehicle or ibrutinib (30 mg/kg) five times per week between weeks 7 and 12 an OGTT was performed one week prior to termination C) and quantified D). Plasma insulin E) and non‐fasted blood glucose F) were measured at termination. Data were analysed by a one‐way ANOVA followed by a Bonferroni *post‐hoc* test and the mean is expressed mean± SEM. *P < 0.05.
Figure S2: Ibrutinib treatment reduces inflammation in the diabetic kidney via inhibition of NF‐kB and the NLRP3 inflammasome.
Figure S3: A) Assessment of cytotoxicity of ibrutinib with MTT assay in RAW blue cells. B) Representative flow cytometry plots of BMDM differentiation at day 7. C) Relative gene expression of *IL‐1*b*, IL‐18, IL‐6 and TNF*a were assessed by qPCR and normalized to 18S. *P < 0.05.
