# Supplementary material for: Inhibition of Bruton's TK regulates macrophage NF‐κB and NLRP3 inflammasome activation in metabolic inflammation
Source: Br J Pharmacol. 2020 Aug 26;177(19):4416–32. doi: 10.1111/bph.15182 (PMC7484557; doi:10.1111/bph.15182)
Supplement: Supplementary file 1 — Figure S1: A) C57BL/6 mice, fed a standard diet (chow) or a high‐fat diet (HFD) for 12 weeks and OGTT was performed at week 6 and week 12 and quantified B). C57BL/6 mice, fed a standard diet (chow) were treated with vehicle or ibrutinib (30 mg/kg) five times per week between weeks 7 and 12 an OGTT was performed one week prior to termination C) and quantified D). Plasma insulin E) and non‐fasted blood glucose F) were measured at termination. Data were analysed by a one‐way ANOVA followed by a Bonferroni post‐hoc test and the mean is expressed mean± SEM. *P < 0.05. Figure S2: Ibrutinib treatment reduces inflammation in the diabetic kidney via inhibition of NF‐kB and the NLRP3 inflammasome. Figure S3: A) Assessment of cytotoxicity of ibrutinib with MTT assay in RAW blue cells. B) Representative flow cytometry plots of BMDM differentiation at day 7. C) Relative gene expression of IL‐1b, IL‐18, IL‐6 and TNFa were assessed by qPCR and normalized to 18S. *P < 0.05. [file BPH-177-4416-s001.pdf]

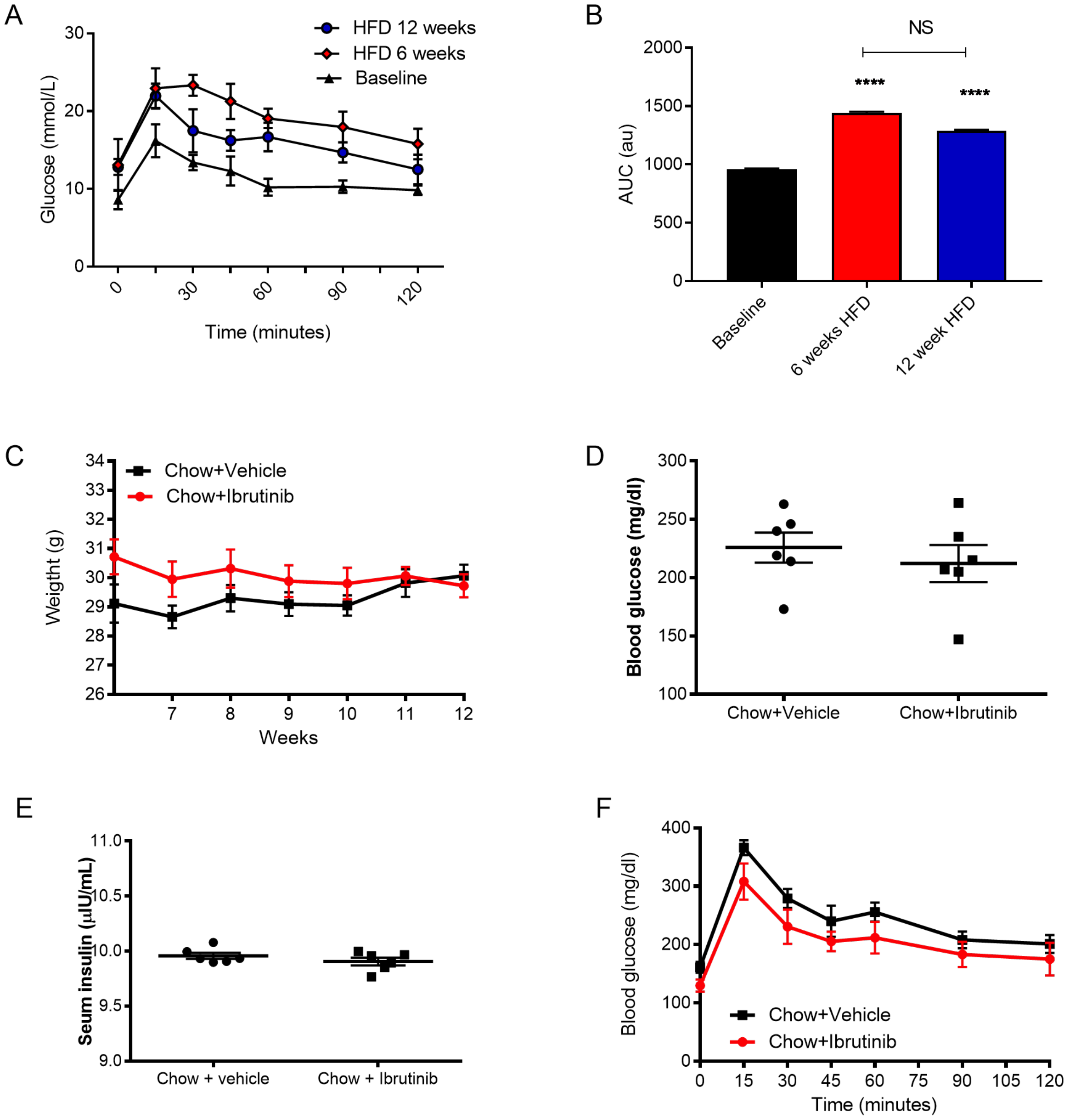

Supplementary Figure 1:

A) C57BL/6 mice, fed a standard diet (chow) or a high-fat diet (HFD) for 12 weeks and OGTT was performed at week 6 and week 12 and quantified B). C57BL/6 mice, fed a standard diet (chow) were treated with vehicle or ibrutinib (30 mg/kg) five times per week between weeks 7 and 12 an OGTT was performed one week prior to termination C) and quantified D). Plasma insulin E) and non-fasted blood glucose F) were measured at termination. Data were analysed by a one-way ANOVA followed by a Bonferroni *post-hoc* test and the mean is expressed mean± SEM. $*p < 0.05$.

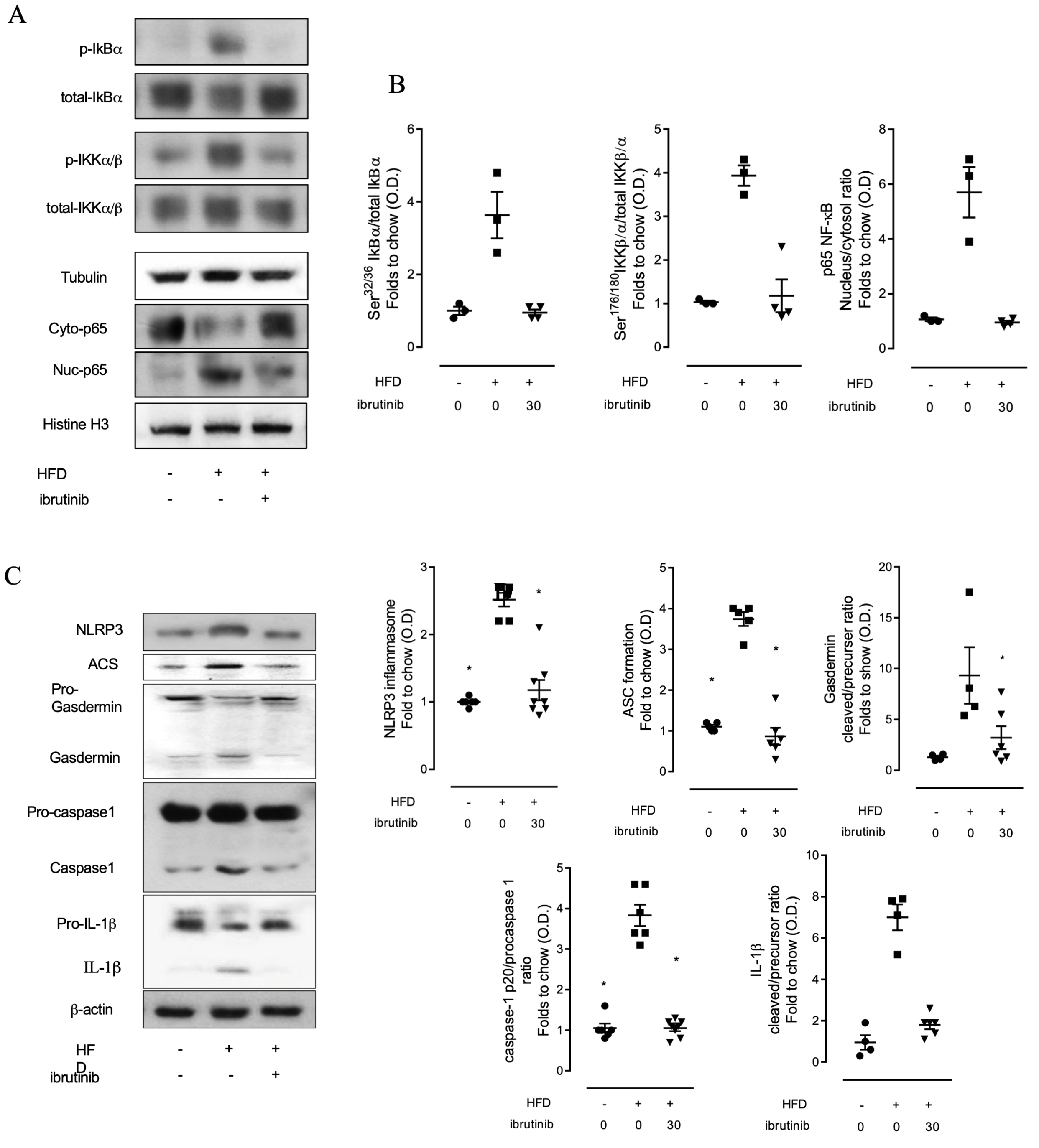

Supplementary Figure 2: Ibrutinib treatment reduces inflammation in the diabetic kidney via inhibition of NF-κB and the NLRP3 inflammasome.

C57BL/6 mice, fed a standard diet (chow) or a high-fat diet (HFD) for 12 weeks, were treated with vehicle or ibrutinib (3 or 30 mg/kg) five times per week between weeks 7 and 12. A) Representative western blots for phosphorylation of $Ser^{32/36}$ on IKBα in the liver and normalized to total IKBα; for phosphorylation of

Ser$^{176/180}$ on IKKα in the liver and normalized to total IKKα; nuclear translocation of p65 and B) quantified using densitometry. C) Representative western blots for NLRP3 inflammasome assembly, ASC, formation of gasdermin, the proteolytic cleavage of pro-caspase 1 to caspase 1 and formation of IL-1β normalized to β-actin and D) quantified using densitometry. Western blots (n=3-6 per group) were analysed by a one-way ANOVA followed by a Bonferroni *post-hoc* test and the mean is expressed mean± SEM. *$p < 0.05$ vs. HFD + Veh.

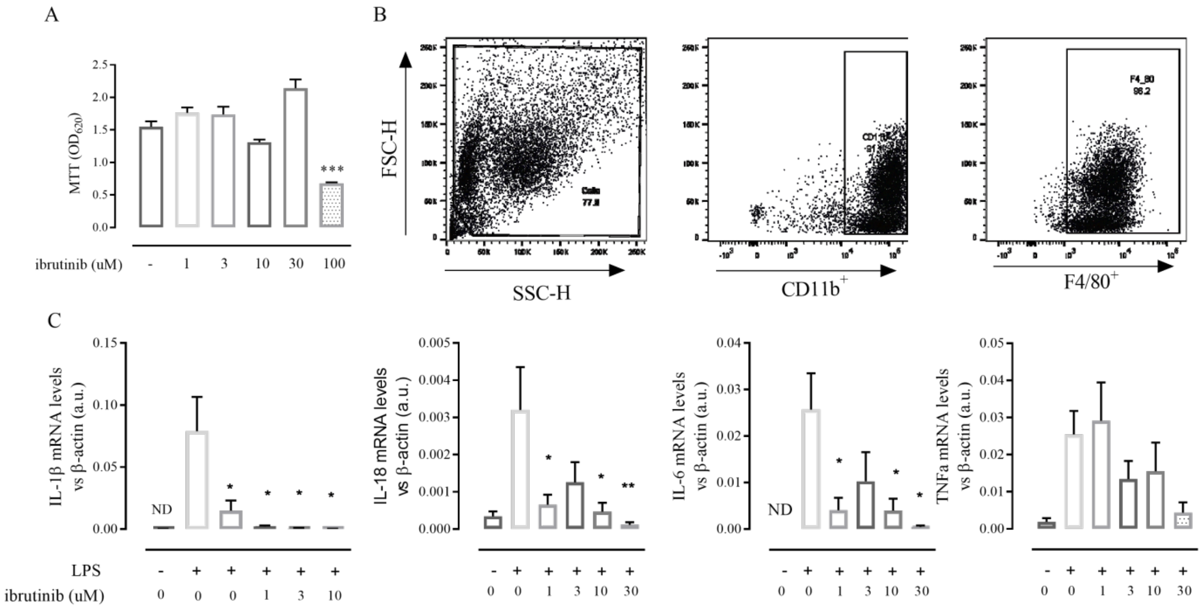

Supplementary Figure 3:

A) Assessment of cytotoxicity of ibrutinib with MTT assay in RAW blue cells. B) Representative flow cytometry plots of BMDM differentiation at day 7. C) Relative gene expression of *IL-1β, IL-18, IL-6 and TNFα* were assessed by qPCR and normalized to 18S. *p<0.05.
